# Country-Level Analysis of the Association between Maternal Obesity and Neonatal Mortality in 34 Sub-Saharan African Countries

**DOI:** 10.5334/aogh.2510

**Published:** 2019-12-06

**Authors:** Ifeoma D. Ozodiegwu, Hadii M. Mamudu, Liang Wang, Richard Wallace, Megan Quinn, Ying Liu, Henry V. Doctor

**Affiliations:** 1 Institute for Global Health, Feinberg School of Medicine, Northwestern University, Chicago, Illinois, US; 2 Department of Health Services Management and Policy, East Tennessee State University Johnson City, Tennessee, US; 3 Department of Biostatistics and Epidemiology, East Tennessee State University, Johnson City, Tennessee, US; 4 Quillen College of Medicine Library, East Tennessee State University Johnson City, Tennessee, US; 5 Department of Science, Information and Dissemination, World Health Organization, Regional Office for the Eastern Mediterranean, Cairo, EG

## Abstract

**Background:**

Prior work examining the association of maternal obesity and neonatal mortality indicate the presence of a positive relationship. However, regional evidence to provide insight on country-level heterogeneities within sub-Saharan Africa (SSA) with nationally representative datasets are non-existent.

**Objective:**

We aimed to determine the relationship between maternal obesity and neonatal mortality at the country level in SSA countries. Moreover, we also estimate regional measures of association to complement previous findings.

**Methods:**

Demographic and Health Survey (DHS) data from 34 SSA countries conducted from 2006–2016 were used for this study. After missing data (36.9% of cases) were addressed with multiple imputations, we identified a total of 175,860 women for the analysis. Complete case and multiply imputed datasets were analyzed individually with multilevel logistic regression models. Potential confounders adjusted for in the regression model included maternal age, level of educational attainment, area of residence, access to prenatal care, birth order and multiple birth (singleton vs twin birth). Regional and country-specific associations were computed, and unadjusted and adjusted odds ratios (ORs), along with the confidence intervals (CIs) were reported.

**Findings:**

Of the total study population, 8,451 (7.6%) were obese. In the regional level analyses, maternal obesity was associated with 40% increased odds of neonatal deaths. This finding was consistent in subgroup analyses by urban and rural residence, and geographic region of residence in SSA. Additionally, obese women were more likely to report neonatal death in the first week of life (OR, days 0–1: 1.39, 95% CI 1.15–1.69; OR, days 2–6: 1.35, 95% CI 1.02–1.79). In the individual country analyses, majority of the countries studied had central estimates supporting elevated odds of neonatal mortality, but the confidence intervals were imprecise.

**Conclusion:**

This study highlights the potential burden of neonatal mortality borne by obese women in SSA. There is, however, a need for longitudinal studies to confirm the results.

## Introduction

Regional evidence highlights changes in adiposity and the looming problem of obesity in Sub-Saharan Africa (SSA) [1,2,3]. An analysis of 245 population-based surveys of 1.2 million participants aged 18 years and older conducted from 1980 to 2014 in 50 African countries found that among women, the age-standardized mean body mass index (BMI) increased by 3 kg/m^2^ [1]. Similarly, Neupane et al. found in a study of 32 African countries that the prevalence of obesity among women in seven African countries were in the double digits [3]. Growing maternal adiposity trends coupled with an inadequate health care system could potentially exacerbate the large burden of neonatal mortality in SSA, which currently stands at 40% of the global estimate [4].

The decline of neonatal mortality in SSA has been slow, compared to other regions globally [4]. This phenomenon means that understanding the influence of maternal obesity on neonatal mortality is imperative for developing country-specific maternal and child health interventions to meet the 2030 Sustainable Development Goal (SDG) 3, Target 3.2, of reducing neonatal mortality to at least as low as 12 neonatal deaths per 1,000 live births [5]; and Every Newborn Action Plan 2035 target of less than 10 neonatal deaths per 1,000 live births [6], therefore, warranting this study.

Existing epidemiological evidence provide support for a positive association between maternal adiposity with neonatal mortality. The best evidence for this relationship is a 2014 meta-analysis of twelve cohort studies, which discovered that every five unit increase in BMI, assessed before or in early pregnancy, was associated with a 15% increased risk of neonatal mortality [7]. In the same study, the absolute risk of neonatal deaths per 10,000 pregnancies, for obese mothers in the BMI categories of 30–40 kg/m^2^, ranged from 24–34 compared to only 20 neonatal deaths per 10,000 pregnancies for normal-weight women with a BMI of 20 and 22.5 kg/m^2^ [7]. It has been postulated that the relationship between maternal obesity and early neonatal mortality may be mediated by diabetic and hypertensive disorders [8]. Nonetheless, the generalizability of these findings to SSA is unknown because all the aforementioned studies arose from Western countries. Cresswell and colleagues’ cross-sectional analysis of the association of maternal obesity and neonatal mortality in 27 countries within 2003 to 2009 is the only SSA study conducted with a nationally representative dataset [9]. Cresswell’s study found a 46% elevated odds of neonatal mortality among obese women [9]. However, additional analysis to provide disaggregated data on country-specific associations remain unavailable, potentially impacting national level policy making and intervention planning. This is because regional level analysis may mask any existing heterogeneities at the country-level.

Moreover, given that Cresswell et al. used participants’ post-pregnancy BMI (3 months or more after gestation) at the time of the survey as a proxy for pre-pregnancy BMI in their study, it is imperative that their analysis is reproduced with more recent samples to determine the consistency of their findings and strengthen the knowledge base of this issue. Lastly, Cresswell et al. did not indicate how they dealt with missing data in their study, although BMI was only measured in a sub-sample of participants in DHS surveys from some countries [10]. Ignoring the sample with missing BMI data could lead to biased estimates if their study characteristics differ from that of included participants. Hence, this study complements the existing literature by analyzing more recent DHS data at the regional and country level using both complete case and missing data analytic methods.

## Methods

### Data

We utilized recently conducted DHS data from 34 countries in SSA, which are publicly available from www.dhsprogram.com. We constructed two pooled datasets of responses from adult women, 20–49 years, one containing complete cases after list-wise deletion and the other containing both complete and missing cases (whole dataset). Pregnant women, women less than three months post- partum and participants less than 20 years were excluded. In particular, participants less than 20 years were excluded because they were likely to experience rapid changes in weight due to puberty [11,12]. In addition, to lower the possibility of recall bias, women whose children were born more than five years from the survey were excluded from the sample while nulliparous women were also excluded because they did not form part of the study population. The Supplementary Appendix (Table 1) contains a list of all included surveys by country and year and each individual survey response rate.

The outcome variable, neonatal mortality, which is defined as death within 28 days of life, was categorized as “yes” and “no”. The main independent variable was maternal BMI, categorized as “underweight”, “optimum”, “overweight”, and “obese”. Table 1 presents the outcome variable, exposure variable, covariates, and their categories with a description of how these categories were created.

**Table 1:** Study variable description.

Variable	Original Data Format	Analysis Variable Format

***Outcome Variable***		
Neonatal Mortality	Continuous – Age at death in days, months or years	If response was ≤28 days then neonatal mortality was categorized as “Yes” or 1, otherwise, neonatal mortality = “No” or 0.
***Exposure Variable***		
*Maternal Body Mass Index (BMI) Categories	Continuous – BMI was calculated and recorded post survey from measured weight (W) and height (H) (BMI = W/H^2^)	<18.5 kg/m^2^ was categorized as underweight, 18.5–24.99 kg/m^2^ was categorized as optimum, 25–29.99 kg/m^2^ was categorized as overweight, ≥30 kg/m^2^ was categorized as obese.
***Covariates***		
Maternal Age at Index Birth	Absent in the original dataset.	Dates in the dataset was stored as century month codes (CMC), which is the number of months since the start of the century). To obtain maternal age at the time of the index birth, the CMC of date of birth of the index child was subtracted from the CMC of date of birth of the respondent and divided by 12 to give the maternal age at the time of index birth.
Maternal Education Level	No education Primary education Secondary Higher educatio	No education Primary education Secondary or higher education
Area of Residence	Urban Rural	Urban Rural
Access to Prenatal Care	The type of person who gave prenatal care to the respondent prior to birth was recorded as: Doctor Nurse/Midwife No one Traditional Birth Attendant Country-specific health professional (several categories)	Access to prenatal care was defined as care provided by skilled health care personnel [13]. These are typically Doctors, Nurses and Midwives [14]. Hence, access to prenatal care was categorized as “Yes” or 1 if care was provided by a Doctor, Nurse or Midwife, otherwise “No” or 0.
Birth Order	Discrete – birth order number of the child which gives the order in which the child was born	Same as original data format
Multiple Births	0 – single birth 1 and upward – multiple birth	No or 0 – single birth Yes or 1 – multiple birt
Region	Absent in the dataset	This variable was created by categorizing the country of the respondent into regional classification following the method of the UN Statistics Division [15] East Africa West Africa Middle Africa Southern Africa
Time Interval since Index Birth (years)	Absent in the dataset	The CMC of the date of interview was subtracted from the CMC of the date of birth of child to provide the time interval since the index birth.

A key assumption in our study was that each participant’s current BMI was representative of their BMI prior to their last pregnancy. Based on existing literature [16,9], we selected the following confounders – maternal age, maternal education level, area of residence, access to prenatal care, birth order and multiple births – for inclusion in the analysis. Covariates such as region and time interval since index birth were included in the study to adjust for any distributional differences that could confound the results of the study.

### Statistical analysis

Descriptive analyses of complete cases were conducted to produce frequencies and percentages of participants by reports of neonatal mortality and within categories of maternal BMI and covariates. Initial analysis of the whole dataset with missing cases focused on examining the missing data patterns and identifying the percentage of missing observations per variable. Analysis of the missing data patterns indicated that a missing completely at random assumption was inappropriate, hence, missing cases were imputed with a fully conditional specification method [17]. Overall and subgroup estimates of neonatal mortality were calculated in the complete case and multiply imputed datasets.

Subsequently, we conducted a multilevel logistic regression to estimate the magnitude of the association, in the form of odds ratios (ORs), between maternal BMI, included covariates, and neonatal mortality using both complete and multiply imputed datasets. Multilevel models were formulated using the mixed effects modelling procedure where data have been collected in nested units. Sampling cluster was included in the model as nested random effects with country modelled as fixed effects. The general specification of the model is presented in equation (1):

1 logit\;\left({{\pi _{ijk}}} \right) = \log \left({\frac{{{\pi _{ijk}}}}{{1 - {\pi _{ijk}}}}} \right) = {\beta _0} + {X_{ijk}} + \;{u_{0jk}}

where π*_ijk_* is the probability of dying for the *i*th neonate in *j*th cluster and in *k*th country, *X_ijk_* is a vector of covariates corresponding to the *i*th mother in the *j*th cluster and *k*th country, β_0_ is a vector of unknown parameters, *u*_0_*_j_* is the random effect at the cluster level. The intercept or average probability of observing neonatal mortality is assumed to vary randomly across women and clusters. With this approach, the fixed effects (measures of association) are expressed as OR with associated 95% confidence intervals (CI).

This approach led to the estimation of unadjusted and adjusted ORs of the likelihood of neonatal death in SSA. Stratified analyses were conducted to further explore the association of maternal BMI with neonatal mortality to estimate the unadjusted and adjusted ORs of neonatal death in various subpopulations – by area of residence (urban women and rural women) and by region of residence (East Africa, West Africa, Middle Africa, Southern Africa). Additionally, individual binary logistic regression models were used to estimate the adjusted ORs for neonatal death within three periods (days 0–1, 2–6, and 7–27) to determine the odds of neonatal death within each discrete period.

Subsequently, individual multilevel logistic regression models of the association between maternal obesity and neonatal mortality were also performed for each country. Due to limited number of observations for the key outcome variables in the analysis, we were not able to determine individual country estimates for Madagascar and Niger for the complete datasets. Similarly, for the multiply imputed datasets, we were not able to get individual country estimates for 6 of the 34 (17.6%) countries: Comoros, Lesotho, Mali, Sao Tome & Principe, Swaziland and Togo. For these countries, there were variables that had many categories (e.g. BMI) with limited observations and led to models that failed to converge. Since BMI categories could not be combined to address the challenges of model convergence, individual estimates for these countries were not obtainable; however, all these countries are included in the pooled analyses. The adjusted ORs of neonatal mortality from the multilevel logistic regression model for each country were used to conduct a meta-analysis to develop a forest plot of the adjusted pooled effect across all the countries examined. The pooled effect focused on obese women compared with women who had optimum weight.

The pooled ORs with associated 95% Cls were estimated using Mantel-Haenszel statistical methods. Heterogeneity among the surveys was assessed using *I^2^* statistics, a measure of the proportion of total variability explained by heterogeneity rather than chance expressed as a percentage [18]. Roughly, an *I^2^* of 0–40% represents no or little heterogeneity, 30–60% moderate heterogeneity, 50–90% substantial heterogeneity, and 75–100% considerable heterogeneity [19]. The meta-analysis applied a fixed effects analytical model due to little heterogeneity among the survey results. Finally, due to the influence of the OR estimates for neonatal mortality in Nigeria and Tanzania on the computation of the regional estimate, sensitivity analyses were conducted by removing the estimates for these two countries and additional forest plots were created. Design weights provided by the DHS were used to weight all results. All study related analysis was computed in Stata version 14 [20].

## Results

### Participants

**Figure 1:**
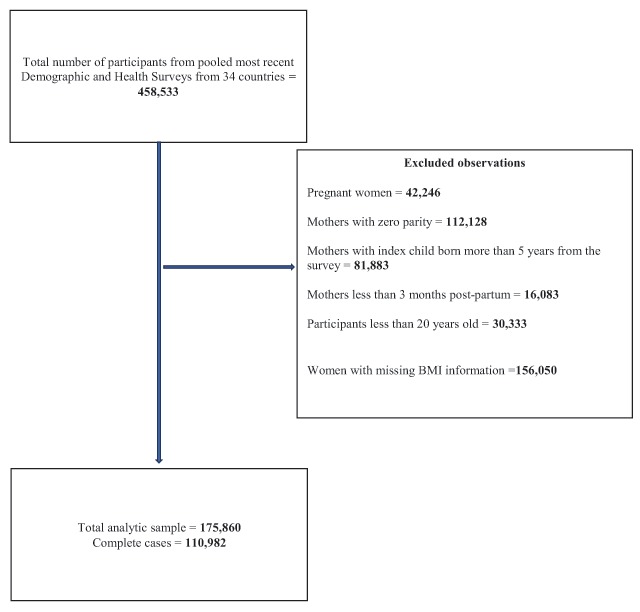
Flow diagram of sample selection.

The pooled dataset contained a total of 458,533 participants. Altogether, we excluded 282,673 observations. We identified 175,860 women for the analysis, of which 110,982 were complete cases and 68,878 (36.9%) had missing data in at least one of the measured variables. Figure 1 presents the initial sample after data pooling, the number of excluded variables, the reasons for exclusion, and the final analytic sample.

Table 2 presents the variables and their respective percentages of missing values.

**Table 2:** Frequencies and percentages of missing values per variable.

Variable	Frequency of missing values (n)	Percent of missing values (%)

Neonatal mortality	64,878	36.9
Body mass index	64,878	36.9
Mothers age at Index Birth	0	0.0
Mother’s education	20	0.0
Place of residence	0	0.0
Access to prenatal care	0	0.0
Birth order	0	0.0
Twin/multiple birth	0	0.0
Region of residence	0	0.0

### Descriptive findings

Table 3 shows the weighted number and percentage distribution of all women by neonatal mortality outcomes and background characteristics for the complete cases. Of the total study sample of women, while 65.3% had optimal BMI, 17.3% were overweight, 9.8% were underweight, and 7.6% were obese. The average age among respondents was roughly 30 years (SD ± 6.3). Table 3 also shows that most women (39.9%) had no formal education and lived in rural areas (66.7%). About 7 out of 10 women (70.2%) had received prenatal care for their most recent birth. On average, participants’ most recent birth was the fourth child and they were mainly single births (97.7%). The highest percentage of women were interviewed in Eastern Africa (41.4%), followed by West Africa (40.7%). The average time interval since index birth was two years (SD ± 1.4). Additional descriptive analysis of the prevalence of obesity by country has been provided in the Supplementary Appendix (Table 2).

**Table 3:** Individual characteristics of women reporting neonatal mortality in 34 SSA countries under complete case analysis.

Characteristics	No (n = 109,140) Number (%)	Yes (n = 1,842) Number (%)	All women (n = 110,982) Number (%)	*P*-value

**Maternal BMI Category**				
Optimum	71,356 [65.4]	1,144 [62.1]	72,501 [65.3]	0.000
Underweight	10,671 [9.8]	164 [8.9]	10,835 [9.8]	
Overweight	18,863 [17.3]	332 [18.0]	19,195 [17.3]	
Obese	8,250 [7.6]	204 [11.0]	8,451 [7.6]	
**Maternal Age at Index Birth (years)**				
Mean (SD*)	29.6 [6.3]	31.5 [6.9]	29.6 [6.3]	
**Maternal Education Level**				
No Education	43,452 [39.8]	791 [43.0]	44,244 [39.9]	0.002
Primary	36,592 [33.5]	629 [34.1]	37,220 [33.5]	
Secondary or Higher	29,082 [26.7]	422 [22.9]	29,504 [26.6]	
**Area of Residence**				
Urban	36,350 [33.3]	615 [33.4]	36,964 [33.3]	0.315
Rural	72,790 [66.7]	1,227 [66.6]	74,018 [66.7]	
**Prenatal Care**				
Yes	76,706 [70.9]	1,172 [63.7]	77,879 [70.2]	0.000
No	32,434 [29.7]	670 [36.4]	33,103 [29.8]	
**Birth Order**				
Mean (SD)	4.2 [2.4]	5.0 [3.0]	4.2 [2.5]	
**Twin/Multiple Birth**				
No	106,855 [97.9]	1,592 [86.4]	108,447 [97.7]	0.000
Yes	2,285 [2.1]	250 [13.6]	2,535 [2.3]	
**Region**				
East Africa	45,187 [41.4]	721 [39.2]	49,508 [41.4]	0.000
West Africa	44,310 [40.6]	826 [44.8]	45,135 [40.7]	
Middle Africa	15,717 [14.4]	236 [12.8]	15,953 [14.4]	
Southern Africa	3,926 [3.6]	59 [3.2]	3,985 [3.6]	
**Time Interval since Index Birth (years)**				
**Mean (SD)**	2.0 [1.4]	2.0 [1.5]	2.0 [1.4]	
**Total**	**109,140 (98.3)**	**1,842 (1.7)**	**110,982 (100.0)**	

* SD = Standard Deviation.

Table 4 presents the design consistent prevalence estimates of neonatal mortality in both the complete case and imputation scenarios for our categorical variables. Overall, both approaches gave a neonatal mortality rate of 17 deaths per 1,000 live births with similar standard errors. Within categories of the exposure variable and covariates, prevalence estimates differed slightly, and CIs overlapped except in the overweight category. Standard errors were generally lower in the multiple imputation datasets. These differences between the multiple imputation and complete case datasets reflect the additional information gained from the imputation process that was ignored during list-wise deletion in the complete case scenario.

**Table 4:** Overall and subgroup estimates of neonatal mortality and their standard errors under (a) complete case analysis and (b) multiple imputation in sub-Saharan Africa.

Variable	(a) Complete case analysis			(b) Multiple imputations		

	Estimate	S.E.	95% CI	Estimate	S.E.	95% CI

**Overall**	**0.017**	**0.0004**	**[0.016, 0.017]**	**0.017**	**0.0003**	**[0.016, 0.017]**

**Maternal BMI Category**						
Optimum	0.016	0.0005	[0.015, 0.017]	0.017	0.0001	[0.017, 0.018]
Underweight	0.015	0.0011	[0.013, 0.017]	0.014	0.0003	[0.014, 0.015]
Overweight	0.017	0.0009	[0.016, 0.019]	0.021	0.0003	[0.020, 0.021]
Obese	0.024	0.0017	[0.020, 0.027]	0.023	0.0004	[0.022, 0.024]
**Maternal Education**						
No Education	0.018	0.0006	[0.017, 0.019]	0.018	0.0005	[0.017, 0.019]
Primary	0.017	0.0007	[0.015, 0.018]	0.016	0.0005	[0.015, 0.017]
Secondary or Higher	0.014	0.0007	[0.013, 0.016]	0.015	0.0006	[0.014, 0.016]
**Area of Residence**						
Urban	0.017	0.0007	[0.016, 0.018]	0.018	0.0006	[0.017, 0.019]
Rural	0.016	0.0005	[0.015, 0.017]	0.016	0.0004	[0.015, 0.017]
**Prenatal Care**						
No	0.020	0.0008	[0.018, 0.021]	0.019	0.0006	[0.018, 0.020]
Yes	0.015	0.0004	[0.014, 0.016]	0.015	0.0004	[0.015, 0.016]
**Multiple Birth**						
No	0.015	0.0004	[0.014, 0.015]	0.015	0.0003	[0.014, 0.015]
Yes	0.097	0.0059	[0.085, 0.108]	0.096	0.0047	[0.087, 0.105]
**Region**						
East Africa	0.015	0.0006	[0.014, 0.016]	0.015	0.0005	[0.014, 0.016]
West Africa	0.019	0.0006	[0.018, 0.020]	0.019	0.0005	[0.017, 0.019]
Middle Africa	0.014	0.0009	[0.012, 0.016]	0.016	0.0008	[0.015, 0.018]
Southern Africa	0.016	0.0020	[0.012, 0.020]	0.016	0.0015	[0.013, 0.019]
**Number of observations**	**110,982**			**175,860**		

### Main Findings

Table 5 presents the unadjusted and adjusted ORs of neonatal mortality for the complete case analysis and multiple imputations at a regional level. The association of maternal BMI category and covariates with neonatal mortality was consistent in both datasets. We report the results from the adjusted analysis for the multiple imputations. Our findings indicate that neonatal mortality increased with increments in maternal BMI. Compared with mothers with optimum BMI, infants born to obese mothers in SSA had an increased likelihood of experiencing neonatal mortality (OR: 1.40, 95% CI: 1.21–1.63). Overweight mothers in SSA also had an elevated likelihood of neonatal mortality (OR: 1.12, 95% CI: 0.99–1.27). Marginal associations with neonatal mortality were found for increasing age (OR: 1.02, 95% CI: 1.01–1.03) and increasing birth order (OR: 1.07, 95% CI: 1.04–1.09). It was found that rural residence and receipt of prenatal care in SSA were associated with an 18% (OR: 0.82, 95% CI: 0.75–0.90) and 24% (OR: 0.76, 95% CI: 0.70–0.83) lower odds of neonatal mortality than urban residence and non-receipt of prenatal care, respectively. Compared to single births, multiple births in SSA had roughly six and half times the odds of neonatal mortality (OR: 6.56, 95% CI: 5.83–7.38).

**Table 5:** Adjusted odds ratios for the multilevel logistic regression models under (a) complete case analysis and (b) multiple imputations in sub-Saharan Africa.

Variable	(a) Complete case analysis		(b) Multiple imputations	

	Crude Odds Ratio (95% CI)	Adjusted Odds Ratio (95% CI)	Crude Odds Ratio (95% CI)	Adjusted Odds Ratio (95% CI)

**Maternal BMI Category**				
Optimum	1.00	1.00	1.00	1.00
Underweight	0.94 [0.79, 1.10]	0.95 [0.81, 1.13]	0.88 [0.76, 1.04]	0.90 [0.77, 1.06]
Overweight	1.12 [0.99, 1.27]	1.09 [0.96, 1.24]	1.15 [1.02, 1.30]	1.12 [0.99, 1.27]
Obese	1.56 [1.33, 1.82]	1.47 [1.25, 1.73]	1.48 [1.28, 1.70]	1.40 [1.21, 1.63]
**Mothers Age at Index Birth (years)**	1.05 [1.04, 1.05]	1.03 [1.02, 1.04]	1.04 [1.04, 1.05]	1.02 [1.01, 1.03]
**Maternal Education Level**				
No Education	1.00	1.00	1.00	1.00
Primary	1.04 [0.92, 1.17]	1.15 [1.01, 1.30]	1.01 [0.92, 1.11]	1.09 [0.99, 1.21]
Secondary or Higher	0.85 [0.74, 0.97]	1.04 [0.89, 1.21]	0.86 [0.77, 0.95]	1.00 [0.89, 1.13]
**Area of Residence**				
Urban	1.00	1.00	1.00	
Rural	0.95 [0.86, 1.05]	0.85 [0.76, 0.95]	0.92 [0.85, 0.99]	0.82 [0.75, 0.90]
**Prenatal Care**				
No	1.00	1.00	1.00	1.00
Yes	0.78 [0.70, 0.87]	0.76 [0.68, 0.85]	0.79 [0.72, 0.86]	0.76 [0.70, 0.83]
**Birth Order**	1.13 [1.12, 1.15]	1.07 [1.04, 1.10]	1.13 [1.11, 1.14]	1.07 [1.04, 1.09]
**Multiple Birth**				
No	1.00	1.00	1.00	1.00
Yes	7.27 [6.31, 8.39]	6.67 [5.76, 7.72]	7.13 [6.35, 7.99]	6.56 [5.83, 7.38]
**Region**				
East Africa	1.00	1.00	1.00	1.00
West Africa	1.18 [0.95, 1.47]	1.13 [0.90, 1.42]	1.17 [0.96, 1.44]	1.11 [0.90, 1.37]
Middle Africa	0.88 [0.66, 1.17]	0.79 [0.59, 1.06]	1.03 [0.79, 1.34]	0.94 [0.72, 1.22]
Southern Africa	1.04 [0.70, 1.55]	1.11 [0.74, 1.66]	1.10 [0.76, 1.57]	1.17 [0.81, 1.69]
**Time Interval since Index birth (years)**	1.00 [0.97, 1.03]	0.95 [0.92, 0.98]	1.01 [0.98, 1.03]	0.96 [0.93, 0.98]
**Number of observations**	**110,982**		**175,840**	

Tables 3–8 of the supplementary appendix reports the results of the stratified analyses in the following subpopulations: urban women, rural women, East African women, West African women, Middle African women and Southern African women. In all our stratified analyses, obese women had increased odds of experiencing neonatal mortality. Specifically, in the analysis by area of residence, urban obese women had a 40% elevated odds of experiencing neonatal mortality (OR: 1.40, 95% CI: 1.15–1.70) and rural obese women had a 41% elevated odds (OR: 1.41, 95% CI: 1.13–1.75). Additionally, in the analyses by geographic region of residence within SSA, East African women had a 48% elevated odds of experiencing neonatal mortality (OR: 1.48, 95% CI: 1.13–1.93), West African women had a 37% elevated odds (OR: 1.37, 95% CI: 1.09–1.73), Middle African women had a 37% elevated odds (OR: 1.37, 95% CI: 0.90–2.07), and Southern African women had a 31% elevated odds (OR: 1.31, 95% CI: 0.71–2.41).

Table 6 presents the ORs for neonatal mortality according to maternal BMI within three discrete periods (0–1, 2–6, and 7–27 days) for the complete case analysis and multiply imputed models. Compared with those born to optimum weight women, children born to obese women had a 39% increased odds of dying during the first two days of life (OR 1.39, 95% CI 1.15–1.69) and a 35% increased odds of dying within the second to the sixth day of life (OR 1.35, 95% CI 1.02–1.79).

**Table 6:** Timing of neonatal mortality under (a) complete case analysis and (b) multiple imputations in sub-Saharan Africa.

Characteristics	Number of deaths	Complete case analysis		Multiple imputations	

		Crude O.R. [95% Cl]	Adjusted O.R. [95% Cl]	Crude O.R. [95% Cl]	Adjusted O.R. [95% Cl]

**Days 0–1**					
Optimum (Ref)	614	1.00	1.00	1.00	1.00
Underweight	84	0.83 [0.66, 1.05]	0.86 [0.68, 1.08]	0.85 [0.69, 1.04]	0.89 [0.73, 1.09]
Overweight	187	1.16 [0.99, 1.37]	1.13 [0.96, 1.34]	1.14 [0.96, 1.36]	1.08 [0.90, 1.28]
Obese	122	1.68 [1.38, 2.05]	1.60 [1.30, 1.97]	1.54 [1.28, 1.85]	1.39 [1.15, 1.69]
**Days 2–6**					
Optimum (Ref)	294	1.00	1.00	1.00	1.00
Underweight	46	1.00 [0.73, 1.37]	1.02 [0.75, 1.40]	0.96 [0.72, 1.27]	0.97 [0.79, 1.49]
Overweight	85	1.08 [0.84, 1.37]	1.04 [0.81, 1.33]	1.10 [0.88, 1.39]	1.02 [0.76, 1.36]
Obese	62	1.59 [1.19, 2.14]	1.48 [1.09, 2.02]	1.43 [1.09, 1.86]	1.35 [1.02, 1.79]
**Days 7–27**					
Optimum (Ref)	227	1.00	1.00	1.00	1.00
Underweight	34	1.18 [0.84, 1.65]	1.19 [0.85, 1.67]	1.06 [0.77, 1.45]	1.08 [0.79, 1.49]
Overweight	58	0.99 [0.74, 1.33]	1.01 [0.75, 1.36]	1.03 [0.78, 1.37]	1.02 [0.76, 1.36]
Obese	17	0.92 [0.60, 1.41]	0.95 [0.61, 1.49]	1.04 [0.70, 1.54]	1.01 [0.67, 1.53]

*Note*: Ref – Reference category.

**Figure 2:**
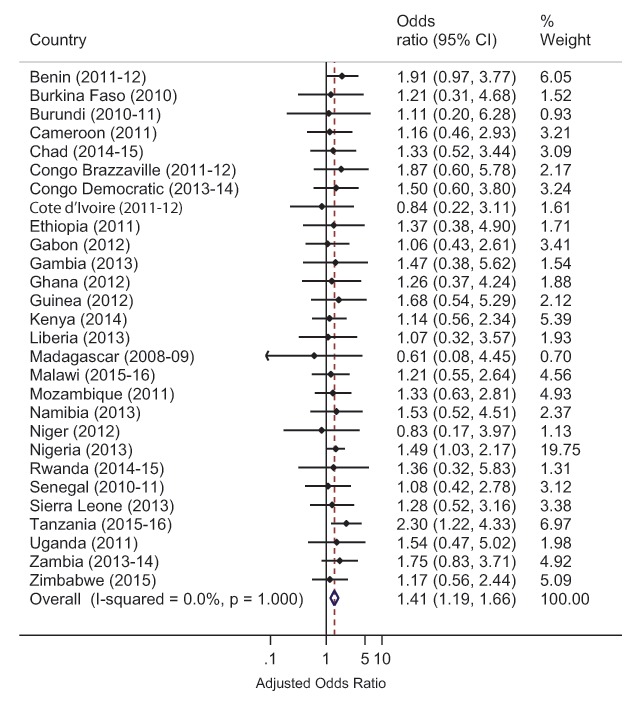
Forest plot of country-level association of maternal obesity and neonatal mortality under imputations.

**Figure 3:**
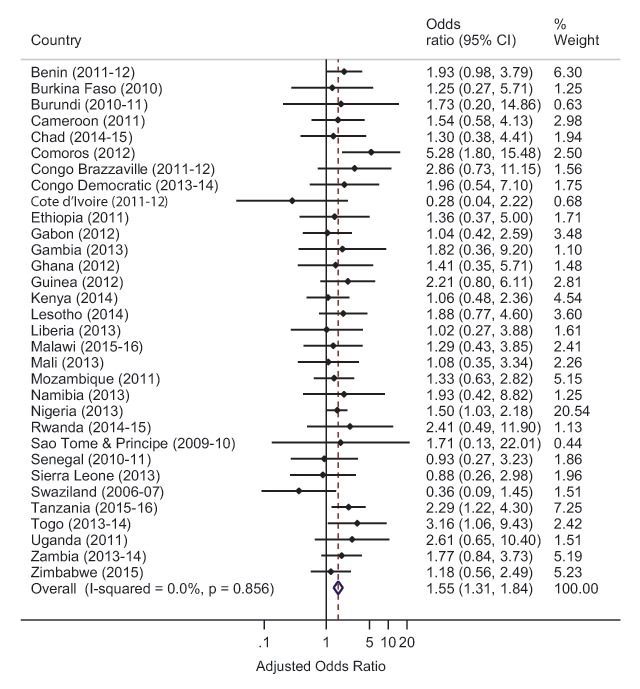
Forest plot of country-level association of maternal obesity and neonatal mortality under complete case analysis.

Disaggregated analyses of the association between maternal obesity and neonatal mortality at the country level were obtainable for 28 countries in the multiply imputed analysis (Figure 2) and for 32 countries in the complete case analysis (Figure 3). In sum, 26 of the 28 countries in the multiply imputed analysis, and 28 of the 32 countries in the complete case analysis had central estimates that tended towards increased odds of neonatal mortality for obese women. The regional estimate for the odds of neonatal mortality in obese women in the forest plot of estimates from the multiply imputed analysis (Figure 2) were similar to the results of regression model (Table 5) (OR 1.40, 95% CI 1.21–1.63 vs OR 1.41, 95% CI 1.19–1.66). The results of the sensitivity analyses of the regional estimate for the odds of neonatal mortality in the forest plots (Figures 2 and 3 in the supplementary appendix) revealed that even in the absence of the estimates from Nigeria and Tanzania, in both the multiply imputed and complete case analysis respectively, obese women in SSA had an elevated odds of neonatal mortality (OR 1.32, 95% CI 1.09–1.60 and OR 1.51, 95% CI 1.24–1.85).

## Discussion

Our study provides detailed analysis of the association between maternal obesity and neonatal mortality at the regional level in SSA while unmasking regional heterogeneities through individual country level analyses. Additionally, our study also presents information on the odds of neonatal mortality in different time periods for obese women [21]. In the pooled analysis, we found a dose-response relationship between maternal BMI and neonatal mortality with obese women exhibiting a 40% increase in the odds of reporting neonatal mortality, with an uncertainty range of 21% to 63% increased odds of neonatal mortality. The central estimate for the elevated odds of neonatal mortality implies that if obese SSA women experience 28 neonatal deaths per 1,000 live births, women of optimal weight will experience approximately eight fewer deaths, with a lower limit of five fewer deaths and an upper limit of 17 fewer deaths, if they were to have the same number of live births. Our findings are especially consequential at the population level where such small differences can create wide disparities in birth outcomes between obese mothers and optimum weight mothers. The elevated odds of neonatal mortality among obese SSA women persisted both in the meta-analysis of odds ratios, even after removal of influential estimates, and in our stratified analyses as there were no differences by urban and rural area of residence, or by geographic region of residence within SSA. Further, our analyses also revealed that the odds of neonatal mortality for obese women, compared to women of optimum weight, was elevated in the first week of life.

Unlike prior regional analyses [9], we addressed issues related to missing data and provided estimates from both our analysis of the complete cases and multiple imputation. Although our findings were consistent with this work [9], it differed in magnitude. Specifically, Cresswell et al. reported 46% increased odds of experiencing neonatal death among obese women in SSA, which was present in the first month of life [9].

Studies outside the SSA region also support the results of our regional analyses. This includes a 2006 analysis of the DHS data from Brazil, which revealed that overweight or obesity in women was associated with an increased odds of death in the neonatal period (OR: 2.41, 95% CI: 1.12–5.16) [22]. However, a similar study with DHS data from nine Asian countries (Bangladesh, India, Maldives, Nepal, Pakistan, Cambodia, Timor-Leste, Kyrgyzstan, and Tajikistan) only found elevated odds of neonatal mortality among overweight women (OR: 1.48, 95% CI: 1.23–1.78) but not among obese women [23]. Nonetheless, the findings of our regional analyses are consistent with the results of a meta-analysis of cohort studies from Western and Asian countries that indicated that maternal obesity was associated with a 15% increased risk of neonatal mortality [24], although the effect estimates from the DHS studies were much higher. The higher effect estimates are likely due to exaggerations associated with the use of an odds ratio [25], residual confounding that often plagues cross-sectional studies [25] or reflect the differential biological susceptibility to obesity-related metabolic conditions, such as preeclampsia and gestational diabetes [26,27,28], that may mediate the relation between maternal obesity and neonatal mortality.

The direction of the central estimates of the individual country analyses support the existence of an elevated odds of neonatal mortality among obese women in majority of the countries under investigation in this work. In particular, we found central estimates indicating elevated odds of neonatal mortality among obese women in 26 of 28 countries analyzed in the multiple imputation scenario, and in 26 of 32 countries analyzed in the complete case scenario. However, due to the small sample sizes of obese women as compared to optimal weight women in the individual country analyses, confidence intervals lacked precision warranting confirmatory studies. Nonetheless, the range of compatible odds ratio estimates suggested by the confidence intervals from most of the individual countries indicate that obese women could potentially have lower odds of neonatal mortality or they could have very high odds of neonatal mortality, compared to optimal weight women. In Nigeria, the central estimate implies that if obese women experience 28 deaths per 1,000 live births, optimal weight women will experience roughly nine fewer deaths with a range of roughly one fewer death to 15 fewer deaths. In Tanzania, using the same example for obese women as Nigeria, it is expected that optimal weight women will experience 15 fewer deaths with a range of roughly five fewer deaths to 21 fewer deaths. Collectively, our findings suggest very wide disparities in neonatal birth outcomes could exist between obese mothers and optimal weight mothers in majority of the examined countries.

In a few countries, however, central estimates indicating a lower odds of neonatal mortality in obese mothers compared to normal weight mothers were found in three countries (Cote d’Ivoire, Madagascar, and Niger) in the multiply imputed analyses and four countries (Cote d’Ivoire, Senegal, Sierra Leone) in the complete case analyses. A potential reason for this finding could be differential selection for obese mothers without comorbid conditions, which may mediate neonatal death. For instance, in their analyses of singleton deliveries from 2005 to 2016 in Switzerland, Aubry and colleagues found that obese mothers without diabetic and hypertensive disorders had a 6% lower risk of neonatal death compared to optimal weight mothers [8]. Nonetheless, these findings warrant further confirmation as the upper limit of confidence intervals in both our work and that of Aubry was in direction of an elevated odds of neonatal mortality in obese mothers.

The need for more confirmatory studies on this topic in SSA is heightened because SSA has one of the highest global neonatal mortality rates (28 deaths per 1,000 live births) and one of the slowest rate of decline, compared to other regions [29]. Moreover, the numbers of neonatal deaths in SSA has increased between 1990 and 2018, and it is projected that 42 of 48 countries in the region will miss the 2030 SDG neonatal mortality target [29]. Simultaneously, overweight and obesity among women of reproductive age in SSA are at an upward trajectory [30,31,32]. Since maternal obesity increases the risk of the leading causes of neonatal death – preterm birth complications [33,34] and intrapartum complications, in particular, preeclampsia – greater comprehension of its contribution to the regional burden of neonatal mortality will feed into the development of national strategic plans and intervention planning.

To more efficiently capture neonatal deaths in order to allow for ease in the specification of temporal relationship between maternal obesity and neonatal mortality, increased investments in vital registration records and services that encourage the uptake of prenatal and delivery services in health-care institutions are needed. Alternatively, ongoing sampling methods such as those developed in India whereby births and deaths are continuously enumerated in sample areas by part-time workers and every six months through independent retrospective surveys can be used [36].

Moreover, in designing new studies on this topic, it is important that the interaction of obese pregnant SSA women with the health system, including health providers, in individual countries are examined as this might also explain the neonatal health outcomes related to maternal obesity. There is some evidence to suggest that obese mothers in SSA might receive sub-optimal care. For instance, a qualitative study of the perceptions of health care workers towards maternal obesity in Nigeria found that participants expressed inadequate knowledge of clinical care practices for obese pregnant women due to the absence of clinical practice guidelines [35].

This study is strengthened by the use of nationally representative data from the included SSA countries [9]. Compared to a previous study [9], our study provides more precise estimates by addressing missing data, incorporating seven more countries, and offering disaggregated analysis at the country-level to guide program and policy action. However, our study should be interpreted in the light of some data limitations. Firstly, our assumption that the BMI of non-pregnant women was similar to their pre-pregnancy BMI after the index birth could have led to a misclassification of exposure with subsequent impact on the effect estimates. Nonetheless, our analysis of the effect of time on BMI only showed a negligible change that was insufficient to lead to misclassification to another BMI category (Supplementary appendix Figure 1). Secondly, self-reports of neonatal deaths expose the study estimates to recall bias which could result to misclassification of still births to neonatal deaths and heaping (preference for reporting deaths to coincide with a particular day, week or month). However, there is no evidence that points to differential reporting and misclassification by weight status. Lastly, the absence of cause of death data for the neonates makes it impossible to determine the plausibility of obesity or related complications as a determinant of death.

In conclusion, this study investigated the association between maternal obesity and neonatal mortality at regional and country levels. Our estimates suggest that obese women may have increased odds of neonatal mortality in most of the SSA countries included in this work. However, longitudinal studies are needed to confirm the observed findings. Nevertheless, precautionary actions are warranted to ensure that obese women have access to quality and available health care before and during childbirth to decrease the burden of neonatal deaths.

## Additional File

The additional file for this article can be found as follows:

10.5334/aogh.2510.s1 Supplement. Includes a description of the systematic search strategy to identify the evidence before this study, and additional analytical tables and plots referenced in the manuscript.
