# Supplementary material for: Country-Level Analysis of the Association between Maternal Obesity and Neonatal Mortality in 34 Sub-Saharan African Countries
Source: Ann Glob Health. 2019 Dec 6;85(1):139. doi: 10.5334/aogh.2510 (PMC6896841; doi:10.5334/aogh.2510)

Supplement

Country-level analysis of the association between maternal obesity with neonatal mortality in sub-Saharan Africa: cross-sectional study of Demographic and Health Surveys from 34 countries

Ifeoma D. Ozodiegwu, DrPH[1], Hadii Mamudu PhD[2], Liang Wang MD, DrPH[3], Richard Wallace EdD[4], Megan Quinn DrPH[3], Ying Liu PhD[3], Henry V. Doctor PhD[5]

[1]Institute for Global Health, Feinberg School of Medicine, Northwestern University, Chicago, Illinois, Tennessee, United States of America
[2]Department of Health Services Management and Policy, East Tennessee State University Johnson City, Tennessee, United States of America
[3]Department of Biostatistics and Epidemiology, East Tennessee State University, Johnson City, Tennessee, United States of America
[4]Quillen College of Medicine Library, East Tennessee State University Johnson City, Tennessee, United States of America
[5]Department of Science, Information and Dissemination, World Health Organization, Regional Office for the Eastern Mediterranean, Cairo, Egypt

Corresponding Author: Ifeoma D. Ozodiegwu

Email: ifeoma.ozodiegwu@northwestern.edu

Phone: +1 423 7731809

Contents

Systematic review search strategy 3

Table 1: Complete details on all included surveys and excluded variables by survey, Demographic and Health Surveys 2006-2016 4

Table 2: Proportion of maternal BMI categories within all 34 included countries (Demographic and Health Surveys) 6

Table 3. Adjusted odds ratios (ORs) for the multilevel logistic regression models under (a) complete case analysis and (b) multiple imputations for urban women 8

Table 4. Adjusted odds ratios (ORs) for the multilevel logistic regression models under (a) complete case analysis and (b) multiple imputations for rural women 9

Table 5. Adjusted odds ratios for the multilevel logistic regression models under (a) complete case analysis and (b) multiple imputations for East African women 10

Table 6. Adjusted odds ratios (ORs) for the multilevel logistic regression models under (a) complete case analysis and (b) multiple imputations for West African women 11

Table 7. Adjusted odds ratios (ORs) for the multilevel logistic regression models under (a) complete case analysis and (b) multiple imputations for Middle African women 12

Table 8. Adjusted odds ratios (ORs) for the multilevel logistic regression models under (a) complete case analysis and (b) multiple imputations for Southern African women 13

Figure 1: effect of time on BMI 14

Figure 2: Forest plot of country-level association of maternal obesity and neonatal mortality under imputations, excluding Nigeria and Tanzania 15

Figure 3: Forest plot of country-level association of maternal obesity and neonatal mortality under complete case analysis, excluding Nigeria and Tanzania 16

Systematic review search strategy

Databases were searched on July 22, 2017, PubMed was searched using the strategy “Neonatal mortality AND Africa AND obesity”. This produced 34 hits. The search strategy was chosen after many unproductive attempts. The search success was measured by its ability to retrieve highly pertinent articles known before the search was attempted. A second PubMed strategy used was "Africa South of the Sahara"[MeSH Terms] AND "Obesity"[MESH] AND ("Infant Mortality"[MESH] OR "Infant, Newborn"[MeSH Terms]). This search produced 25 hits. No limits were used.

The LILACS database was searched with the strategy “neonatal mortality AND obesity AND Africa”. This produced no results. The African Index Medicus Database was searched with the strategy “neonatal mortality AND obesity. Two hundred results were saved. The PROQUEST collection of databases, which included ABI/INFORM , Biology Database , Health & Medical Collection, Health Management Database, Nursing & Allied Health Database, and the Public Health Database was searched using all text except full text with the strategy “Neonatal mortality and obesity and Africa” which resulted in12 hits.

CINAHL (EBSCO interface) was text word searched using the strategy “Neonatal mortality AND obesity AND Africa”. This produced 58 hits. The Cochrane Database of Systematic Reviews and Cochrane CENTRAL (EBSCO interface) were searched by text word using the search phrase “Neonatal mortality and obesity and Africa” producing 24 hits. Web of Science was searched producing eight results using “Neonatal mortality and obesity and Africa” as the search. PsychInfo (EBSCO interface) produced three results using the strategy “Neonatal mortality AND obesity AND Africa”. In total we found 364 citations. Only one study out of these citations investigated the relationship between maternal obesity and neonatal mortality with nationally representative data in sub-Saharan Africa.

The PubMed search was updated on May 8, 2018 and no new article investigating the relationship between maternal obesity and neonatal mortality in sub-Saharan Africa was found.

Table 1: Complete details on all included surveys and excluded variables by survey, Demographic and Health Surveys 2006-2016

				Exclusions							
**Number of Countries**	**Country survey by year**	**Overall Female Response Rate**	**Total number of observations in dataset**	**Pregnant Women**	**Mothers with zero parity**	**Index child born more than five years from survey**	**Mothers less than 3 months post-partum**	**Participants less than 20 years old**	**Missing Observations**	**Total observations left**	**% observations left in dataset**
1	Burkina Faso 2010	97.7	17087	1687	3541	2425	689	1021	3862	3862	22.6
2	Benin 2011 -12	94.4	16599	1595	3781	3115	519	766	145	6678	40.2
3	Burundi 2010 -11	95.5	9389	928	3264	888	315	301	1902	1791	19.1
4	Congo DR 2013-14	98.5	18827	2404	4181	2440	906	1353	3724	3819	20.3
5	Congo 2011-12	97.8	10819	1114	1822	1928	425	993	2117	2420	22.4
6	Cote d'Ivoire 2011-12	91	10060	1016	2331	1820	404	695	1961	1833	18.2
7	Cameroon 2011	96.4	15426	1492	4110	3048	519	1130	2433	2694	17.5
8	Ethiopia 2011	93.2	15683	1122	5183	2706	467	733	195	5277	33.6
9	Gabon 2012	97.5	8422	873	1836	2070	289	684	846	1824	21.7
10	Ghana 2014	95.9	9396	679	2750	1866	255	352	1718	1776	18.9
11	Gambia 2013	86.2	10233	852	3215	1338	444	548	2070	1766	17.3
12	Guinea 2012	97.4	9142	967	2003	1613	319	827	1633	1780	19.5
13	Kenya 2014	95.6	31079	2076	7377	7177	874	1642	6262	5671	18.2
14	Comoros 2012	89.7	5329	340	2332	788	133	174	30	1532	28.7
15	Liberia 2013	97.1	9239	834	1518	1960	322	860	1813	1932	20.9
16	Lesotho 2014	95.7	6621	276	1956	1658	167	461	1014	1089	16.4
17	Madagascar 2008-09	94.4	17375	1449	4036	3921	562	1418	3066	2923	16.8
18	Mali 2012-13	94.4	10424	1165	1774	1429	472	935	2262	2387	22.9
19	Malawi 2015-16	96.9	24562	1833	5030	4607	717	2212	6741	3422	13.9
20	Mozambique 2011	98.9	13745	1409	2794	2599	501	1164	60	5218	38.0

Table 1: Complete details on all included surveys and excluded variables by survey, Demographic and Health Surveys 2006-2016

				Exclusions							
**Number of Countries**	**Country survey by year**	**Overall Female Response Rate**	**Total number of observations in dataset**	**Pregnant Women**	**Mothers with zero parity**	**Index child born more than five years from survey**	**Mothers less than 3 months post-partum**	**Participants less than 20 years old**	**Missing Observations**	**Total observations left**	**% observations left in dataset**
21	Nigeria 2013	96.7	38948	4493	10635	6402	1381	2006	175	13856	35.6
22	Niger 2012	93.5	11160	1479	1747	1332	637	790	2816	2359	21.1
23	Namibia 2013	89.4	10018	586	2527	2996	245	547	1627	1490	14.9
24	Rwanda 2014-15	99.4	13497	954	4520	2393	330	358	2501	2441	18.1
25	Sierra Leone 2013	96.5	16658	1426	3949	3085	537	1436	3123	3102	18.6
26	Senegal 2010-11	91.2	15688	1297	4779	2189	619	978	3707	2119	13.5
27	Sao Tome and Principe 2008-09	84.5	2615	225	555	480	89	168	94	1004	38.4
28	Swaziland 2006-07	89.6	4987	273	1409	1206	115	456	41	1487	29.8
29	Chad 2014-15	95.1	17719	2460	3233	2687	873	1446	2451	4569	25.8
30	Togo 2013-14	96.8	9480	811	2368	1689	263	417	1969	1963	20.7
31	Tanzania 2015-16	95.7	13266	1137	3279	2226	490	836	34	5264	39.7
32	Uganda 2011	89.4	8674	963	2098	1260	389	531	2327	1106	12.8
33	Zambia 2013-14	94.2	16411	1420	3677	2584	547	1399	64	6720	40.9
34	Zimbabwe 2015	95.1	9955	611	2518	1958	269	696	95	3808	38.3
		**94.5**	**458533**	**42246**	**112128**	**81883**	**16083**	**30333**	**64878**	**110982**	**24.2**

Table 2: Proportion of maternal BMI categories within all 34 included countries (Demographic and Health Surveys)

#	Country	*n*	Underweight (<18.5kg/m2)	%	Normal (18.5-24.9kg/m2)	%	Overweight (25-29.9kg/m2)	%	Obese (≥30kg/m2)	%
1	Burkina Faso [2010]	3,879	554	14.3	2,925	75.4	294	7.6	106	2.7
2	Benin [2011-12]	6,647	303	4.6	4,444	66.9	1,435	21.6	465	7
3	Burundi [2010-11]	1,849	252	13.6	1,453	78.6	108	5.9	36	1.9
4	Congo DR [2013-14]	3,806	502	13.2	2,666	70.1	509	13.4	129	3.4
5	Congo [2011-12]	2,237	288	12.9	1,304	58.3	425	19	221	9.9
6	Cote d'Ivoire [2011-12]	1,770	86	4.8	1,222	69.1	349	19.4	119	6.7
7	Cameroon [2011]	2,713	189	7	1,582	58.3	607	22.4	334	12.3
8	Ethiopia [2011]	5,732	1,207	21.1	4,117	71.8	327	5.7	81	1.4
9	Gabon [2012]	1,691	71	4.2	789	46.7	458	27.1	372	22
10	Ghana [2014]	1,713	86	5	862	50.3	478	27.9	286	16.7
11	Gambia [2013]	1,736	234	13.5	1,082	62.3	303	17.4	118	6.8
12	Guinea [2012]	1,771	164	9.2	1,249	70.5	273	15.4	86	4.9
13	Kenya [2014]	5,516	454	8.2	3,132	56.8	1,332	24.2	599	10.9
14	Comoros [2012]	1,563	64	4.1	737	47.2	490	31.3	272	17.4
15	Liberia [2013]	1,743	104	6	1,136	65.2	349	20	154	8.9
16	Lesotho [2014]	1,075	31	2.9	520	48.4	295	27.4	229	21.3
17	Madagascar [2008-09]	2,984	842	28.2	1,966	65.9	147	4.9	30	1
18	Mali [2012-2013]	2,400	226	9.4	1,712	71.3	342	14.2	122	5.1
19	Malawi [2015-2016]	3,419	186	5.5	2,462	72	564	16.5	206	6
20	Mozambique [2011]	5,319	356	6.7	4,145	77.9	633	11.9	184	3.5
21	Nigeria [2013]	13,994	1,233	8.8	8,948	63.9	2,698	19.3	1,115	8

Table 2: Proportion of maternal BMI categories within all 34 included countries (Demographic and Health Surveys)										
#	**Country**	***n***	**Underweight (<18.5kg/m2)**	**%**	**Normal (18.5-24.9kg/m2)**	**%**	**Overweight (25-29.9kg/m2)**	**%**	**Obese (≥30kg/m2)**	**%**
22	Niger [2012]	2,353	296	12.6	1,599	67.9	354	15.1	104	4.4
23	Namibia [2013]	1,419	147	10.4	768	54.2	296	20.8	208	14.7
24	Rwanda [2014-15]	2,460	126	5.1	1,789	72.7	452	17.5	145	4.7
25	Sierra Leone [2013]	3,144	252	8	2,294	73	446	14.4	143	4.6
26	Senegal [2010-11]	2,097	376	17.9	1,216	58	366	17.5	140	6.7
27	Sao Tome and Principe [2008-09]	919	41	4.5	525	57.2	236	25.7	116	12.7
28	Swaziland [2006-07]	1,491	18	1.18	585	39.2	480	32.2	409	27.4
29	Chad [2014-15]	4,587	793	17.3	3,243	70.7	448	9.8	103	2.3
30	Togo [2013-14]	1,886	114	6.1	1,188	63	378	20	206	10.9
31	Tanzania [2015-16]	5,234	387	7.4	3,327	63.6	989	18.9	531	10.1
32	Uganda [2011]	1,091	112	10.3	771	70.7	157	14.4	50	4.6
33	Zambia [2013-14]	6,770	594	8.8	4,494	66.4	1,180	17.4	502	7.4
34	Zimbabwe [2015]	3,971	150	3.8	2,247	56.6	1,019	25.7	556	14
	**Pooled Data**	**110,982**	**10,835**	**9.8**	**72,501**	**65.3**	**19,195**	**17.3**	**8,452**	**7.6**

Table 3. Adjusted odds ratios (ORs) for the multilevel logistic regression models under (a) complete case analysis and (b) multiple imputations for urban women

Variable	**(a) Complete case analysis**		**(b) Multiple imputations**	
	**Crude ORs (95% CI)**	**Adjusted ORs (95% CI)**	**Crude ORs (95% CI)**	**Adjusted ORs (95% CI)**
**Maternal BMI category**				
Optimum	1.00	1.00	1.00	1.00
Underweight	1.01 [0.72, 1.42]	1.00 [0.71, 1.41]	0.97 [0.68, 1.38]	0.95 [0.67, 1.36]
Overweight	0.97 [0.80, 1.19]	0.96 [0.78, 1.17]	1.03 [0.85, 1.25]	1.05 [0.86, 1.27]
Obese	1.48 [1.19, 1.84]	1.40 [1.12, 1.76]	1.39 [1.15, 1.69]	1.40 [1.15, 1.70]
**Mothers age at birth (years)**	1.05 [1.04, 1.07]	1.03 [1.01, 1.05]	1.04 [1.03, 1.05]	1.03 [1.01, 1.04]
**Maternal education**				
No education	1.00	1.00	1.00	1.00
Primary	0.88 [0.71, 1.09]	1.01 [0.81, 1.27]	0.88 [0.74, 1.04]	0.95 [0.79, 1.14]
Secondary or higher	0.62 [0.50, 0.77]	0.81 [0.64, 1.04]	0.64 [0.54, 0.75]	0.73 [0.61, 0.89]
**Prenatal care**				
No	1.00	1.00	1.00	1.00
Yes	0.71 [0.58, 0.86]	0.74 [0.60, 0.91]	0.70 [0.60, 0.82]	0.70 [0.59, 0.83]
**Birth order**	1.16 [1.13, 1.20]	1.06 [1.01, 1.12]	1.13 [1.10, 1.16]	1.03 [0.99, 1.07]
**Multiple birth**				
No	1.00	1.00	1.00	1.00
Yes	6.81 [5.32, 8.74]	6.23 [4.84, 8.04]	6.33 [5.16, 7.77]	6.00 [4.87, 7.40]
**Region**				
East Africa	1.00	1.00	1.00	1.00
West Africa	1.38 [1.00, 1.89]	1.22 [0.89, 1.67]	1.18 [0.91, 1.52]	1.04[0.80, 1.34]
Middle Africa	1.14 [0.77, 1.70]	1.00 [0.68, 1.48]	1.25 [0.91, 1.72]	1.15 [0.84, 1.58]
Southern Africa	0.85 [0.45, 1.61]	0.94 [0.50, 1.77]	1.72 [0.57, 1.51]	1.01 [0.62, 1.65]
**Time interval since index birth (years)**	1.00 [0.95, 1.06]	0.96 [0.91, 1.01]	0.97 [0.93, 1.01]	0.93 [0.89, 0.97]
**Number of observations**	**36,482**		**56,248**	

Table 4. Adjusted odds ratios (ORs) for the multilevel logistic regression models under (a) complete case analysis and (b) multiple imputations for rural women

Variable	(a) Complete case analysis		(b) Multiple imputations	
	**Crude ORs (95% CI)**	**Adjusted ORs (95% CI)**	**Crude ORs (95% CI)**	**Adjusted ORs (95% CI)**
**Maternal BMI category**				
Optimum	1.00	1.00	1.00	1.00
Underweight	0.91 [0.75, 1.09]	0.94 [0.77, 1.13]	0.86 [0.73, 1.02]	0.90 [0.75, 1.06]
Overweight	1.24 [1.06, 1.46]	1.18 [1.00, 1.39]	1.23 [1.06, 1.44]	1.17 [1.00, 1.37]
Obese	1.63 [1.28, 2.06]	1.50 [1.18, 1.92]	1.52 [1.23, 1.87]	1.41 [1.13, 1.75]
**Mothers age at birth (years)**	1.05 [1.04, 1.05]	1.03 [1.01, 1.04]	1.05 [1.04, 1.05]	1.02 [1.01, 1.03]
**Maternal education**				
No education	1.00	1.00	1.00	1.00
Primary	1.08 [0.93, 1.24]	1.20 [1.04, 1.40]	1.05 [0.93, 1.18]	1.17 [1.04, 1.32]
Secondary or higher	1.00 [0.84, 1.20]	1.30 [1.07, 1.5	0.99 [0.85, 1.15]	1.29 [1.09, 1.51]
**Prenatal care**				
No	1.00	1.00	1.00	1.00
Yes	0.80 [0.70, 0.91]	0.77 [0.68, 0.88]	0.79 [0.72, 0.88]	0.78 [0.70, 0.87]
**Birth order**	1.13 [1.11, 1.16]	0.77 [1.03, 1.10]	1.13 [1.11, 1.14]	1.08 [1.05, 1.11]
**Multiple birth**				
No	1.00	1.00	1.00	1.00
Yes	7.55 [6.34, 8.98]	6.96 [5.84, 8.31]	7.58 [6.60, 8.71]	6.90 [5.98, 7.96]
**Region**				
East Africa	1.00	1.00	1.00	1.00
West Africa	1.14 [0.88, 1.48]	1.14 [0.86, 1.51]	1.19 [0.93, 1.53]	1.18 [0.91, 1.54]
Middle Africa	0.79 [0.56, 1.13]	0.72 [0.50, 1.04]	0.94 [0.68, 1.30]	0.85 [0.60, 1.19]
Southern Africa	1.16 [0.73, 1.86]	1.14 [0.70, 1.87]	1.18 [0.76, 1.82]	1.19 [0.75, 1.87]
**Time interval since index birth (years)**	1.00 [0.96, 1.04]	0.95 [0.91, 0.99]	1.02 [0.99, 1.06]	0.97 [0.94, 1.00]
**Number of observations**	**74,487**		**119,592**	

Table 5. Adjusted odds ratios for the multilevel logistic regression models under (a) complete case analysis and (b) multiple imputations for East African women

Variable	(a) Complete case analysis		(b) Multiple imputations	
	**Crude ORs(95% CI)**	**Adjusted ORs (95% CI)**	**Crude ORs (95% CI)**	**Adjusted ORs (95% CI)**
**Maternal BMI category**				
Optimum	1.00	1.00	1.00	1.00
Underweight	0.78 [0.59, 1.03]	0.77 [0.58, 1.02]	0.77 [0.60, 1.00]	0.77 [0.60, 1.00]
Overweight	1.09 [0.88, 1.35]	1.06 [0.85, 1.31]	1.16 [0.95, 1.41]	1.11 [0.91, 1.37]
Obese	1.74 [1.35, 2.24]	1.63 [1.25, 2.12]	1.58 [1.23, 2.03]	1.48 [1.13, 1.93]
**Mothers age at birth (years)**	1.06 [1.04, 1.07]	1.05 [1.03, 1.07]	1.05 [1.04, 1.06]	1.05 [1.03, 1.06]
**Maternal education**				
No education	1.00	1.00	1.00	1.00
Primary	1.05 [0.87, 1.28]	1.19 [0.97, 1.45]	1.07 [0.91, 1.26]	1.19 [1.00, 1.41]
Secondary or higher	0.85 [0.68, 1.08]	1.00 [0.76, 1.32]	0.88 [0.72, 1.07]	1.00 [0.80, 1.25]
**Area of residence**				
Urban	1.00	1.00	1.00	1.00
Rural	0.94 [0.80, 1.12]	0.83 [0.68, 1.00]	0.89 [0.77, 1.02]	0.78 [0.67, 0.92]
**Prenatal care**				
No	1.00	1.00	1.00	1.00
Yes	0.69 [0.57, 0.82]	0.67 [0.55, 0.80]	0.72 [0.61, 0.84]	0.69 [0.58, 0.81]
**Birth order**	1.14 [1.11, 1.17]	1.03 [0.99, 1.08]	1.13 [1.11, 1.16]	1.03 [0.99, 1.07]
**Multiple birth**				
No	1.00	1.00	1.00	1.00
Yes	7.82 [6.15, 9.94]	7.43 [5.80, 9.51]	6.93 [5.65, 8.51]	6.63 [5.37, 8.18]
**Time interval since index birth (years)**	0.99 [0.94, 1.04]	0.93 [0.88, 0.98]	0.98 [0.94, 1.02]	0.93 [0.89, 0.97]
**Number of observations**	**45,163**		**68,440**	

Table 6. Adjusted odds ratios (ORs) for the multilevel logistic regression models under (a) complete case analysis and (b) multiple imputations for West African women

Variable	(a) Complete case analysis		(b) Multiple imputations	
	**Crude ORs(95% CI)**	**Adjusted ORs (95% CI)**	**Crude ORs (95% CI)**	**Adjusted ORs (95% CI)**
**Maternal BMI category**				
Optimum	1.00	1.00	1.00	1.00
Underweight	1.06 [0.83, 1.34]	1.10 [0.86, 1.40]	0.98 [0.78, 1.22]	1.01 [0.81, 1.27]
Overweight	1.18 [0.99, 1.41]	1.16 [0.97, 1.39]	1.17 [0.99, 1.38]	1.15 [0.97, 1.37]
Obese	1.45 [1.14, 1.84]	1.38 [1.08, 1.78]	1.42 [1.14, 1.77]	1.37 [1.09, 1.73]
**Mothers age at birth (years)**	1.04 [1.03, 1.05]	1.01 [1.00, 1.03]	1.04 [1.03, 1.05]	1.01 [1.00, 1.03]
**Maternal education**				
No education	1.00	1.00	1.00	1.00
Primary	1.10 [0.92, 1.32]	1.20 [0.99, 1.44]	1.05 [0.91, 1.22]	1.15 [0.99, 1.34]
Secondary or higher	0.85 [0.71, 1.03]	1.06 [0.86, 1.32]	0.89 [0.76, 1.04]	1.11 [0.93, 1.32]
**Area of residence**				
Urban	1.00	1.00	1.00	1.00
Rural	0.99 [0.86, 1.15]	0.93 [0.79, 1.09]	1.00 [0.77, 0.95]	0.93 [0.81, 1.06]
**Prenatal care**				
No	1.00	1.00	1.00	1.00
Yes	0.86 [0.74, 0.99]	0.84 [0.72, 0.99]	0.83 [0.73, 0.94]	0.80 [0.71, 0.92]
**Birth order**	1.13 [1.11, 1.16]	1.09 [1.05, 1.13]	1.13 [1.11, 1.15]	1.08 [1.05, 1.12]
**Multiple birth**				
No	1.00	1.00	1.00	1.00
Yes	7.56 [6.18, 9.23]	6.85 [5.59, 8.41]	8.12 [8.12, 9.52]	7.41 [6.29, 8.72]
**Time interval since index birth (years)**	1.02 [0.97, 1.07]	0.97 [0.92, 1.02]	1.02 [0.98, 1.06]	0.97 [0.94, 1.01]
**Number of observations**	**45,410**		**72,657**	

Table 7. Adjusted odds ratios (ORs) for the multilevel logistic regression models under (a) complete case analysis and (b) multiple imputations for Middle African women

Variable	(a) Complete case analysis		(b) Multiple imputations	
	**Crude ORs (95% CI)**	**Adjusted ORs (95% CI)**	**Crude ORs (95% CI)**	**Adjusted ORs (95% CI)**
**Maternal BMI category**				
Optimum	1.00	1.00	1.00	1.00
Underweight	0.98 [0.65, 1.48]	0.99 [0.66, 1.50]	0.88 [0.62, 1.26]	0.90 [0.63, 1.29]
Overweight	1.06 [0.73, 1.54]	0.99 [0.68, 1.46]	1.17 [0.84, 1.63]	1.10 [0.78, 1.55]
Obese	1.65 [1.06, 2.58]	1.49 [0.94, 2.38]	1.50 [1.01, 2.24]	1.37 [0.90, 2.07]
**Mothers age at birth (years)**	1.05 [1.03, 1.07]	1.02 [0.99, 1.05]	1.04 [1.02, 1.05]	1.01 [0.99, 1.03]
**Maternal education**				
No education	1.00	1.00	1.00	1.00
Primary	0.81 [0.58, 1.13]	0.81 [0.58, 1.12]	0.77 [0.61, 0.98]	0.74 [0.59, 0.93]
Secondary or higher	0.76 [0.53, 1.09]	0.78 [0.53, 1.14]	0.72 [0.55, 0.93]	0.66 [0.50, 0.87]
**Area of residence**				
Urban	1.00	1.00	1.00	1.00
Rural	0.73 [0.56, 0.96]	0.62 [0.46, 0.84]	0.71 [0.58, 0.86]	0.59 [0.48, 0.74]
**Prenatal care**				
No	1.00	1.00	1.00	1.00
Yes	0.81 [0.61, 1.07]	0.78 [0.58, 1.04]	0.81 [0.66, 0.99]	0.80 [0.65, 0.98]
**Birth order**	1.14 [1.09, 1.19]	1.10 [1.02, 1.18]	1.12 [1.08, 1.16]	1.09 [1.03, 1.15]
**Multiple birth**				
No	1.00	1.00	1.00	1.00
Yes	4.73 [3.03, 7.37]	4.23 [2.69, 6.66]	4.99 [3.65, 6.83]	4.46 [3.24, 6.14]
**Time interval since index birth (years)**	0.97 [0.88, 1.07]	0.93 [0.85, 1.03]	1.04 [0.97, 1.11]	1.00 [0.93, 1.07]
**Number of observations**	**16,330**		**27,995**	

Table 8. Adjusted odds ratios (ORs) for the multilevel logistic regression models under (a) complete case analysis and (b) multiple imputations for Southern African women

Variable	(a) Complete case analysis		(b) Multiple imputations	
	**Crude ORs (95% CI)**	**Adjusted ORs (95% CI)**	**Crude ORs (95% CI)**	**Adjusted ORs (95% CI)**
**Maternal BMI category**				
Optimum	1.00	1.00	1.00	1.00
Underweight	1.51 [0.52, 4.44]	1.61 [0.54, 4.79]	1.17 [0.41, 3.36]	1.19 [0.41, 3.45]
Overweight	0.84 [0.44, 1.60]	0.84 [0.44, 1.63]	0.92 [0.50, 1.68]	0.95 [0.51, 1.75]
Obese	1.27 [0.69, 2.34]	1.23 [0.65, 2.36]	1.26 [0.70, 2.25]	1.31 [0.71, 2.41]
**Mothers age at birth (years)**	1.01 [0.97, 1.05]	1.02 [0.96, 1.08]	1.01 [0.98, 1.04]	1.01 [0.97, 1.06]
**Maternal education**				
No education	1.00	1.00	1.00	1.00
Primary	4.54 [0.60, 34.16]	4.98 [0.65, 38.07]	1.82 [0.63, 5.24]	1.87 [0.64, 5.45]
Secondary or higher	3.61 [0.49, 26.54]	4.31 [0.56, 33.09]	1.42 [0.51, 3.99]	1.57 [0.54, 4.59]
**Area of residence**				
Urban	1.00	1.00	1.00	1.00
Rural	1.38 [0.78, 2.43]	0.39 [0.76, 2.54]	1.18 [0.77, 1.80]	1.13 [0.72, 1.77]
**Prenatal care**				
No	1.00	1.00	1.00	1.00
Yes	0.66 [0.36, 1.20]	0.68 [0.35, 1.34]	0.73 [0.44, 1.20]	0.61 [0.36, 1.06]
**Birth order**	1.04 [0.92, 1.17]	0.94 [0.78, 1.14]	1.05 [0.96, 1.15]	0.99 [0.85, 1.14]
**Multiple birth**				
No	1.00	1.00	1.00	1.00
Yes	9.37 [3.90, 22.47]	10.22 [3.99, 26.20]	6.26 [2.85, 13.75]	6.20 [2.76, 13.93]
**Time interval since index birth (years)**	1.06 [0.90, 1.24]	1.03 [0.86, 1.22]	0.93 [0.82, 1.05]	0.88 [0.77, 1.01]
**Number of observations**	**4,066**		**6,748**	

Figure 1: Effect of time on BMI

Optimum weight
BMI = 21.48396 + (0.003503 * index since birth)
R-squared = 0.0011
p-value for BMI on index = 0.004

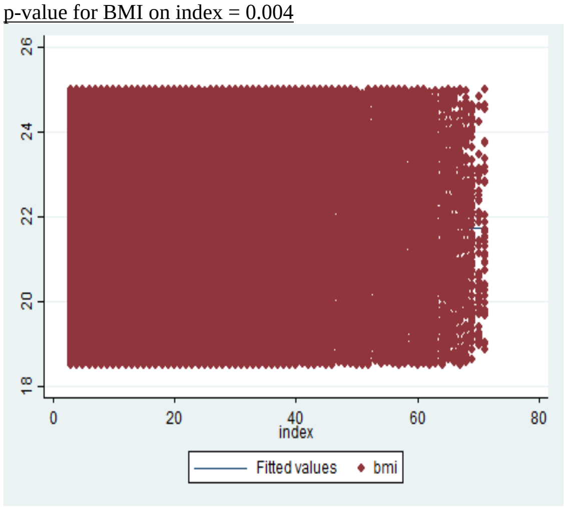

Underweight
BMI = 17.41418 - (0.0009923 * index since birth)
R-squared = 0.0003
p-value for BMI on index = 0.232

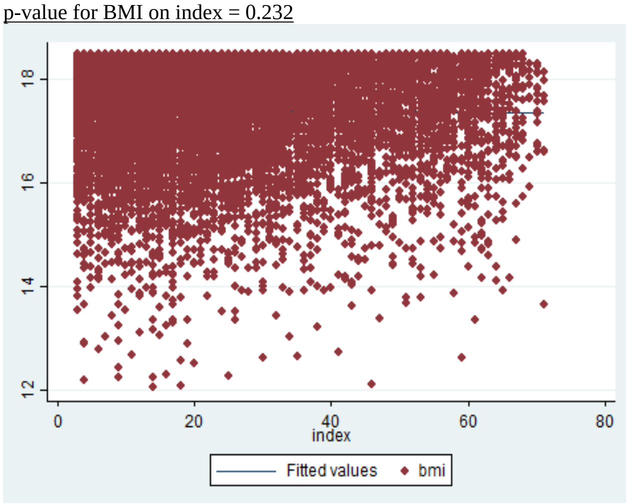

Overweight
BMI = 26.87282 + (0.0043771 * index since birth)
R-squared = 0.0031
p-value for BMI on index = 0.000

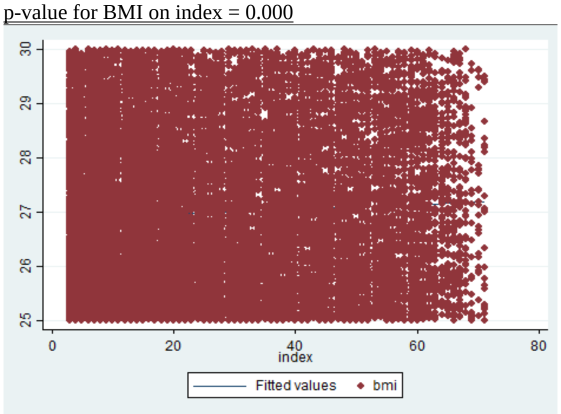

Obese
BMI = 33.74991 + (0.0078104 * index since birth)
R-squared = 0.0012
p-value for BMI on index = 0.007

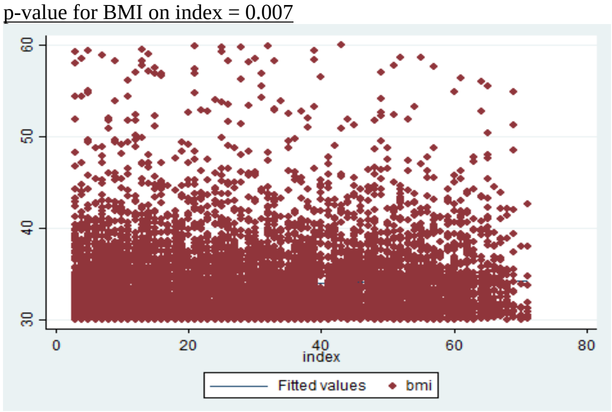

Figure 2: Forest plot of country-level association of maternal obesity and neonatal mortality under imputations, excluding Nigeria and Tanzania

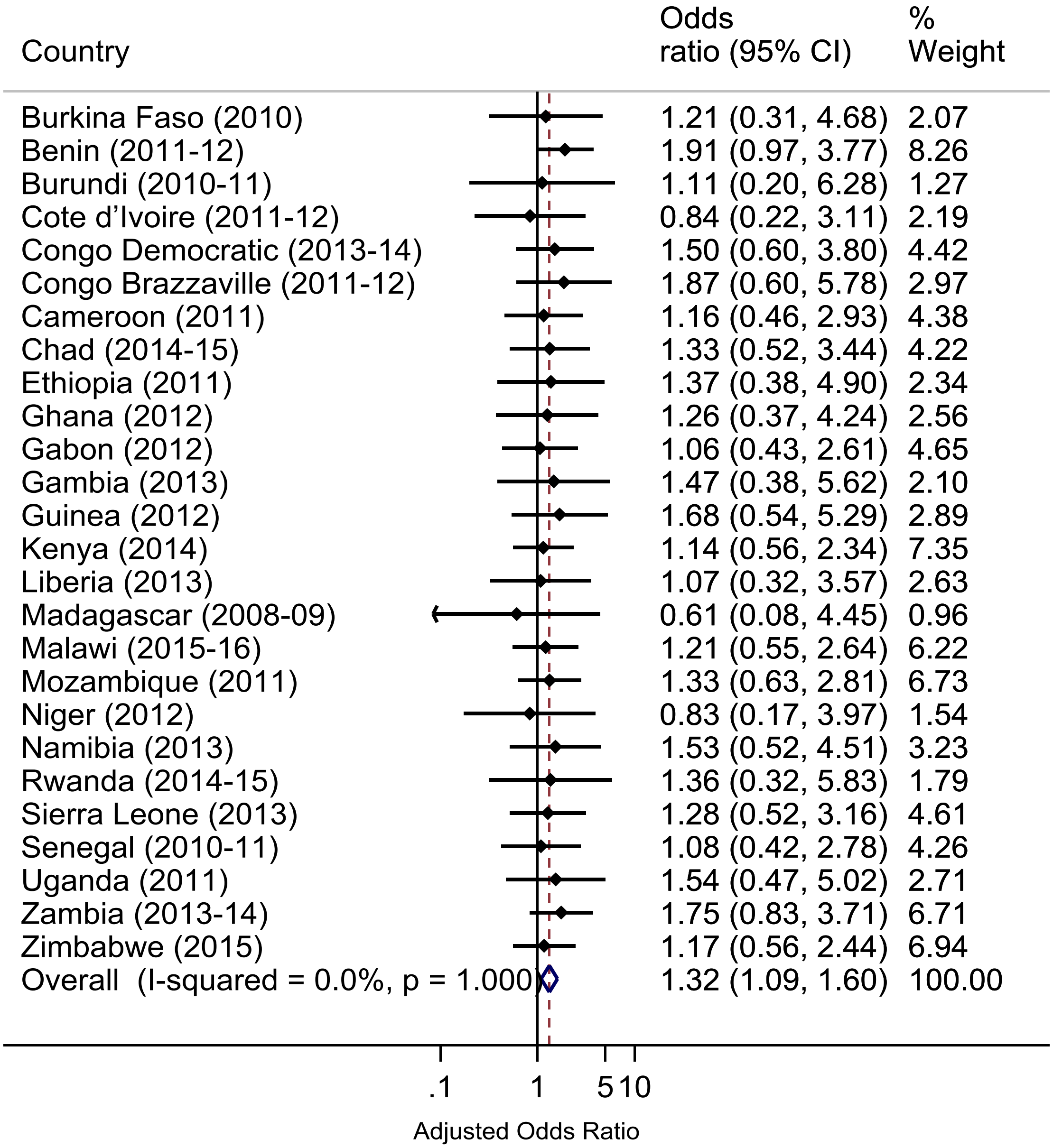

Figure 3: Forest plot of country-level association of maternal obesity and neonatal mortality under complete case analysis, excluding Nigeria and Tanzania

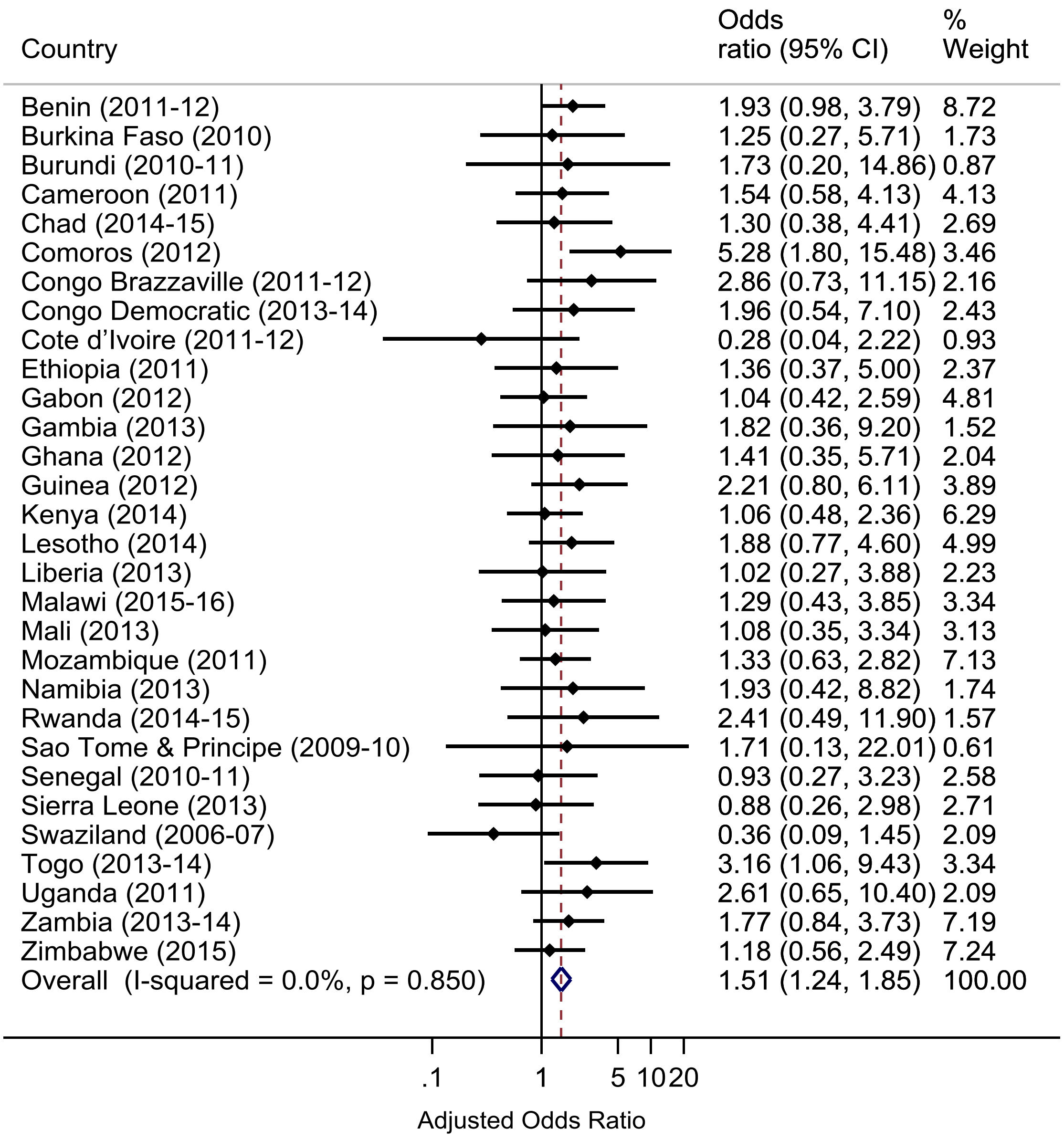

Supplement: Supplement. — Includes a description of the systematic search strategy to identify the evidence before this study, and additional analytical tables and plots referenced in the manuscript. [file agh-85-1-2510-s1.pdf]
